# Common variants in mismatch repair genes associated with increased risk of sperm DNA damage and male infertility

**DOI:** 10.1186/1741-7015-10-49

**Published:** 2012-05-17

**Authors:** Guixiang Ji, Yan Long, Yong Zhou, Cong Huang, Aihua Gu, Xinru Wang

**Affiliations:** 1 State Key Laboratory of Reproductive Medicine, Institute of Toxicology, Nanjing Medical University, Nanjing, 210029, China; 2 Key Laboratory of Modern Toxicology of Ministry of Education, School of Public Health, Nanjing Medical University, Nanjing, 210029, China; 3 Nanjing Institute of Environmental Sciences/Key Laboratory of Pesticide Environmental Assessment and Pollution Control, Ministry of Environmental Protection, Nanjing 210042, China; 4 China Pharmaceutical University, Department of Pharmacology, Nanjing 210024, China; 5 Institute of Health Sciences, Shanghai Institutes for Biological Sciences, Chinese Academy of Sciences & Shanghai Jiao Tong University School of Medicine

## Abstract

**Background:**

The mismatch repair (MMR) pathway plays an important role in the maintenance of the genome integrity, meiotic recombination and gametogenesis. This study investigated whether genetic variations in MMR genes are associated with an increased risk of sperm DNA damage and male infertility.

**Methods:**

We selected and genotyped 21 tagging single nucleotide polymorphisms (SNPs) in five MMR genes (*MLH1, MLH3, PMS2, MSH4 *and *MSH5*) using the SNPstream 12-plex platform in a case-control study of 1,292 idiopathic infertility patients and 480 fertile controls in a Chinese population. Sperm DNA damage levels were detected with the Tdt-mediated dUTP nick end labelling (TUNEL) assay in 450 cases. Fluorescence resonance energy transfer (FRET) and co-immunoprecipitation techniques were employed to determine the effects of functional variants.

**Results:**

One intronic SNP in *MLH1 *(rs4647269) and two non-synonymous SNPs in *PMS2 *(rs1059060, Ser775Asn) and *MSH5 *(rs2075789, Pro29Ser) seem to be risk factors for the development of azoospermia or oligozoospermia. Meanwhile, we also identified a possible contribution of *PMS2 *rs1059060 to the risk of male infertility with normal sperm count. Among patients with normal sperm count, *MLH1 *rs4647269 and *PMS2 *rs1059060 were associated with increased sperm DNA damage. Functional analysis revealed that the *PMS2 *rs1059060 can affect the interactions between MLH1 and PMS2.

**Conclusions:**

Our results provide evidence supporting the involvement of genetic polymorphisms in MMR genes in the aetiology of male infertility.

## Background

Infertility remains a major clinical problem that occurs in 10 to 15% of couples worldwide [1], and male factor infertility accounts for 40 to 50% of all infertility cases [2]. Although several causes have been identified for impaired male fertility [3], the aetiology remains unknown in nearly half of all cases. Currently, a large amount of attention is being paid to the potential effects of sperm DNA damage on male infertility [4]. DNA damage in the male germ line appears as a risk factor for adverse clinical outcomes, including poor semen quality, low fertilization rates, impaired pre-implantation development, miscarriage and an increased risk of morbidity in the offspring [5-7].

Although the clinical significance of testing sperm DNA integrity has been clearly emphasized, the origin of DNA damage in spermatozoa is poorly understood. One mechanism is that deficits in the DNA repair system during spermatogenesis can have negative effects on the integrity of sperm DNA [8,9]. Our previous data have provided strong evidence that some genetic polymorphisms in genes involved in DNA repair were associated with the development of sperm DNA damage and male infertility [10-13].

Among all DNA repair mechanisms, DNA mismatch repair (MMR) plays a critical role in the maintenance of genetic integrity and malfunctions can lead to various cancers in mammals [14-16]. Studies of gene knockout mice indicate that several members of the MMR family also participate in the meiotic recombination process and are involved in gametogenesis [17,18]. Three MutL homologues (MLH1, MLH3 and PMS2) and two MutS homologues (MSH4 and MSH5) are involved in this process.

Based on their important physiological functions, these five MMR genes are good candidate genes for explaining male infertility. Recently, analysis of polymorphic markers in candidate genes helped us to understand the etiology and the susceptibility of male infertility [19-21]. The purpose of this work is three-fold: (1) to examine whether MMR gene polymorphisms are associated with increased risk of azoospermia or oligozoospermia, (2) to ascertain whether genetic variants in MMR genes result in sperm DNA damage and, thereby, increase male infertility, and (3) to investigate the biological activity of the significant functional variants.

## Methods

### Subjects and sample collection

The study was approved by the Ethics Review Board of the Nanjing Medical University. All the studies involving human subjects were conducted in full compliance with government policies and the Declaration of Helsinki. A total of 1,657 infertile patients, diagnosed with unexplained male factor infertility, were drawn from the Centre of Clinical Reproductive Medicine between April 2005 and March 2009 (NJMU Infertile Study). All participants completed an informed consent and a questionnaire, including detailed information, such as age, cigarette smoking, alcohol drinking, tea and vitamin consumption, and abstinence time. All patients underwent at least two semen analyses, and those with a history of orchitis, obstruction of the vas deferens, chromosomal abnormalities, or micro-deletions of the azoospermia factor region on the Y chromosome were excluded [22]. In the final analysis, 1,292 idiopathic infertility patients aged 24 to 42 years old were included, and were divided into three subgroups: 268 infertility patients with non-obstructive azoospermia, 256 infertility patients with oligozoospermia (sperm counts < 20 × 10^6^/ml) and 768 infertility patients with normal count (sperm counts ≥ 20 × 10^6^/ml).

The control group included 480 fertile men ranging from 25 to 40 years of age who had fathered at least one child without assisted reproductive technologies and had normal semen parameters. The semen analysis for sperm concentration, motility and morphology was performed following the World Health Organization criteria [23].

### SNP selection and genotyping

We selected the tagging SNPs by using genotype data obtained from unrelated Han Chinese individuals from Beijing in the HapMap project (HapMap Data Rel 24/Phase II Nov08, on NCBI B36 assembly, dbSNP b126). To examine the gene extensively, we searched the MMR genes, including 2,000 bp of the flanking regions both upstream and downstream of the gene, using the pairwise option of the Haploview 4.0 software (Mark Daly's Lab, Broad Institute of MIT and Harvard, Cambridge, Massachusetts, USA). The tagging SNPs were selected on the basis of pairwise linkage disequilibrium with a r^2 ^threshold of 0.8 and minor allele frequency ≥ 0.05 to capture all the common SNPs. In total, 19 SNPs were chosen in these 5 genes. In addition, a non-synonymous SNP (rs1799977) in *MLH1 *and a non-synonymous SNP (rs2075789) in *MSH5 *that cause missense mutations were included.

Genotyping was performed using TaqMan 7900HT Sequence Detection System and GenomeLab SNPstream high-throughput 12-plex genotyping platform (Beckman Coulter, Fullerton, CA, USA). Sequences of forward, reverse and extension primers are listed in Additional file 1 (Table S1). For quality control, the genotyping was done without knowledge of case/control status of the subjects, and a random 5% of cases and controls were genotyped twice by different individuals, and the reproducibility was 100%. To confirm the genotyping results, selected PCR-amplified DNA samples (n = 2, for each genotype) were examined by DNA sequencing and the results were also consistent.

### DNA fragmentation analysis

After a period of 48 to 72 h of sexual abstinence, semen samples were collected by masturbation into wide-mouthed sterile containers and were delivered to the laboratory within 1 h of ejaculation. The diluted samples were cooled gradually at 5°C for 2 h, frozen at -70°C for Tdt-mediated dUTP nick end labelling (TUNEL) evaluation. A detailed protocol of the TUNEL assay for human sperm has been described previously [24]. TUNEL labeling was carried out using a Cell Death Detection kit (APO-DIRECT kit; BD Biosciences PharMingen, San Diego, CA, USA) according to the manufacturer's instructions. Briefly, semen samples were thawed in a 37°C water bath and immediately diluted with buffer (0.15 M NaCl, 0.01 M Tris, 0.001 M EDTA, pH 7.4) to obtain a sperm concentration of 1 to 2 × 10^6^/ml. Washed sperm was resuspended in 2% paraformaldehyde for 30 minutes at room temperature. After rinsing in PBS, samples were resuspended in permeabilization solution (0.2% Triton X-100, 0.1% sodium citrate) for 10 minutes on wet ice. TUNEL reagent (50 μl) was added to each sample. For each batch, a negative control lacking the terminal deoxynucleotidyl transferase and a positive control treated with DNase I were included to ensure assay specificity. After incubation for 1 h at 37°C, samples were analyzed immediately by flow cytometry (FACSCalibur; BD Biosciences Pharmingen, San Diego, CA, USA). Flow during the analysis was controlled at approximately 500 spermatozoa/sec, and 10,000 cells were analyzed for each sample. The percentage of FITC-positive cells (FL1 channel) was calculated as the percentage of cells with a fluorescence intensity exceeding the threshold obtained with the negative control.

### Plasmid construction

To evaluate the potential effects of *PMS2 *rs1059060 (Ser775Asn) polymorphisms on the interaction between MLH1 and PMS2, fluorescence resonance energy transfer (FRET) technology and immunoprecipitation were performed. The cDNA encoding MLH1 or PMS2 was generated by PCR from a human testis cDNA library.

For the FRET assay, the primers used for amplifying PMS2 (amino acids 655-856) were 5'-CGTTAAGCTTGGAGAAAATCAAGCAGCCGAAG-3'/5'**-**ATACGGATCC CAGGTTGGCGATGTGTCTCAT -3', including *HindIII *and *BamHI *restriction sites (underlined sequences). Point mutations for PMS2 were performed using QuikChange Site-Directed Mutagenesis Kit (Stratagene, La. Jolla, CA, USA). The amplified fragment of PMS2 and its genetic variants were cloned into the pEYFP-C1 vector (Clonetech, Palo Alto, CA, USA). Similarly, the cDNA sequence encoding MLH1 (amino acids 506-756) was amplified by PCR using the following primers: 5'-CGTTGAATTCGTGTTTTGAGTCTCCAGGAAGAAA-3'/5'-ATACGGATCCACACCTCTCAAAGACTTTGTAT-3', which contain *EcoRI *and *BamHI *restriction sites (underlined sequences). This amplified fragment was ligated into pECYP-C1 vector (Clonetech, USA). For immunoprecipitation, the cloning of the full-length PMS2 and MLH1 cDNA constructs into pcDNA3.1 (Invitrogen, Carlsbad, CA, USA), between *NheI *and *BamHI*, has already been described [25]. The integrity of the inserts was confirmed by sequence analysis.

### Cell culture and transfection

MutLα-deficient HEK293T cells were cultured in DMEM: F12 (1:1) (Gibco, Carlsbad, CA, USA), supplemented with 10% foetal bovine serum and 0.1% streptomycin/penicillin (Gibco, USA) in a humidified atmosphere with 5% CO_2 _at 37°C. Cells were seeded onto 30 mm dishes with poly-L-lysine-coated glass coverslips and co-transfected with YFP recombinant plasmid (YFP-PMS2 or variants of YFP-PMS2) and CFP recombinant plasmid (CFP-MLH1) using Lipofectamine 2000 (Invitrogen) until the cells were at 50 to 60% confluence, according to the manufacturer's protocols. The transfection efficiency was compared by Western blotting at 72 hours after transfection using anti-PMS2 (A16-4) (1:100; BD Biosciences), anti-MLH1 (G168-728) (1:100; BD Biosciences), and anti-β actin (1:5000; Santa Cruz Biotechnology, CA, USA) antibodies.

### Image analysis and calculation of fluorescence resonance energy transfer ratios

We used a Zeiss LSM710 confocal microscope (Carl Zeiss, Jena, Germany) operating with a 40 mW argon laser. Filter-cube specifications for the fluorescent channels were as follows for excitation and emission, respectively: enhanced cyan fluorescent protein (ECFP), 430 ± 25 and 470 ± 30 nm; enhanced yellow fluorescent protein (EYFP), 500 ± 20 and 535 ± 30 nm; and fluorescence resonance energy transfer (FRET), 430 ± 25 and 535 ± 30 nm.

Image analysis involved three basic operations: subtraction of background autofluorescence and blurred light, quantification of fluorescence intensity, and calculation of a corrected FRET (FRETc) by the following equation:

*FRETc = (I_DA _- a I_AA _- d I_DD_)/I_AA_*, where *I_DA _*is the fluorescence intensity from the FRET filter set and *I_DD _*and *I_AA _*are the fluorescent intensities from ECFP (the donor) and EYFP (the acceptor), respectively. The cross-talk coefficients *a *and *d *were considered constant. The corrected FRET ratio was defined as FRETc/I_DD_.

### Co-Immunoprecipitation and Western blotting

Proteins were extracted from co-transfected HEK293T cells by the M-PER^® ^Mammalian Protein Extraction Reagent (Pierce Bio, Thermo, Rockford, IL, USA) according to the manufacturer's instruction. Approximately 200 μg total cell protein was transferred to a 1.5 ml microcentrifuge tube, and 20 μl of Protein A/G PLUS-Agarose (Santa Cruz Biotechnology, CA, USA) was added to the supernatant and the mixture was incubated at 4°C on a rocker platform for one hour. After this incubation, 2 μg anti-MLH1 N-20 (Santa Cruz Biotechnology, CA, USA) was added and incubated with shaking at 4°C overnight. The immunoprecipitates were collected by centrifugation at 1,000 × g for 5 minutes at 4°C, washed 4 times with lysis buffer and then the precipitates were collected for the Western blotting detection with the anti-PMS2 (A16-4) (1:100; BD Biosciences) antibody. Proteins were then detected with a Phototope-HRP Western Blot Detection kit (Cell Signalling Technology, Inc., Beverly, MA, USA).

### Statistical analyses

Differences in select demographic variables, as well as smoking and alcohol status, between the cases and the controls, were evaluated using the χ^2 ^test. The Student's *t *test was used to evaluate continuous variables, including age and pack-years of cigarette smoking. The Hardy-Weinberg equilibrium was tested using a goodness-of-fit χ^2 ^test. We used unconditional multivariate logistic regression analysis to examine associations between genetic polymorphisms and male infertility risk by estimating ORs and 95% confidence intervals (95% CI). To reduce the potential for spurious findings due to multiple testing, we applied the False Discovery Rate (FDR) method to the *P-*values for the differences of genotype distributions among cases and controls. False Discovery Rate (FDR) is a new approach to the multiple comparisons problem. Instead of controlling the chance of any false positives (as Bonferroni methods do), FDR controls the expected proportion of false positives among suprathreshold voxels [26].

Sperm DNA fragmentation was normalized by natural logarithm (ln) transformation. Linear regression models were used to estimate the association with ln-transformed sperm fragmentation values for each SNP independently. Models were adjusted for age, smoking status, drinking status and abstinence time. All *P-*values presented are two-sided and all analyses were carried by the Statistical Analysis Software, version 9.1.3 (SAS Institute, Cary, NC, USA).

## Results

### Subject characteristics

The final study population consisted of 1,772 Han Chinese individuals, composed of 480 fertile controls, 268 infertility patients with non-obstructive azoospermia, 256 infertility patients with oligozoospermia (sperm counts < 20 × 10^6^/ml) and 768 infertility patients with normal sperm count (sperm counts ≥ 20 × 10^6^/ml). The frequency distributions of selected characteristics of the case patients and control subjects are presented in Table 1. No significant differences were observed between cases and controls with regard to drinking status or age. However, there was a significantly higher percentage of smokers among cases than controls (*P *< 0.001). Among smokers, cases also reported greater cigarette consumption than controls, as assessed by the mean number of pack-years (*P *< 0.05). As expected, semen parameters, such as sperm concentration and sperm motility, were significantly higher in fertile controls than infertile cases (*P *< 0.001).

**Table 1:** Distribution of selected characteristics between cases and fertile controls

Variables	Controls (n = 480)	**Case 1**^**a**^ (n = 524)		**Case 2**^**b**^ (n = 768)	
			
	N (%)	N (%)	***P***^**d**^	N (%)	***P***^**d**^
Age (mean ± SEM)	28.1 ± 0.16	28.3 ± 0.16	0.374	28.3 ± 0.14	0.355
Smoking stauts					
Never	278 (57.9)	238 (45.4)	< 0.001	363 (47.3)	< 0.001
Ever	202 (42.1)	286 (54.6)		405 (52.7)	
Pack-years (mean ± SEM)^c^	4.3 ± 0.21	5.2 ± 0.20	0.002	4.9 ± 0.14	0.014
Drinking status					
Never	425 (88.5)	447 (85.3)	0.129	667 (86.8)	0.377
Ever	55 (11.5)	77 (14.7)		101 (13.2)	
Semen parameters (mean ± SEM)					
Concentration (× 10^6 ^/ml)	102.6 ± 3.07	5.12 ± 0.38	< 0.001	73.6 ± 2.12	< 0.001
Motility (%)	65.3 ± 0.58	3.26 ± 0.27	< 0.001	37.9 ± 0.55	< 0.001
Volume (ml)	2.80 ± 0.07	2.37 ± 0.07	< 0.001	2.78 ± 0.05	0.818
Sperm DNA fragmentation (%)	n. d.	n. d.		19.5 ± 0.82	

^a^Case 1: idiopathic infertile men with azoospermia or oligozoospermia.

^b^Case 2: idiopathic infertile men with normal sperm count.

^c^Among ever smokers.

^d^*P-*values were derived from the χ^2 ^test for categorical variables (smoking and drinking status) and t test for continuous variables (age and pack-years).

n. d.: not detected.

### Allelic frequencies and genotype distributions of MMR polymorphisms

The position and minor allele frequency among Chinese of the 21 SNPs in the HapMap database are presented in Additional file 2 (Table S2). All SNPs were in Hardy-Weinberg equilibrium among the controls, except for rs3117572 (*P *= 0.024). Inspection of the cluster plots indicated good discrimination between genotypes, suggesting that these deviations from HWE are likely to be chance observations. The genotype distributions among cases and controls are presented in Table 2. Overall, the genotype frequencies of three SNPs were significantly different between the patients with azoospermia or oligozoospermia and the controls (*P *= 0.032 for rs4647269, *P *= 0.003 for rs1059060 and *P *= 0.002 for rs2075789). Moreover, the genotype frequencies of rs1059060 were also significantly different between the patients with normal sperm count and the controls (*P *= 2.0 × 10^-4^).

**Table 2:** Distribution of the genotype in selected SNPs of MMR genes

Gene	tSNP	Controls MAF	**Case1**^**a **^**MAF**	***P***^**c**^	**Case2**^**b**^ MAF	***P***^**c**^
*MLH1*						
	rs1799977	0.023	0.028	0.628	0.025	0.828
	rs4647269	0.047	0.075	**0.032**	0.067	0.175
	rs1540354	0.304	0.343	0.326	0.331	0.428
*PMS2*						
	rs3815383	0.332	0.332	0.923	0.342	0.816
	rs2286680	0.074	0.080	0.772	0.089	0.491
	rs11769380	0.410	0.419	0.816	0.379	0.736
	rs1059060	0.091	0.146	**0.003**	0.170	**2.0 × 10^-4^**
	rs2228006	0.063	0.054	0.520	0.056	0.582
*MLH3*						
	rs13712	0.184	0.185	0.913	0.185	0.962
	rs7156586	0.222	0.217	0.832	0.406	0.527
	rs175049	0.185	0.173	0.646	0.177	0.727
*MSH4*						
	rs1021462	0.309	0.336	0.592	0.317	0.842
*MSH5*						
	rs3749953	0.121	0.154	0.278	0.148	0.653
	rs1150793	0.142	0.141	0.960	0.138	0.827
	rs707939	0.359	0.337	0.582	0.357	0.886
	rs707938	0.304	0.331	0.557	0.336	0.231
	rs3115672	0.375	0.402	0.681	0.377	0.929
	rs3117572	0.223	0.206	0.724	0.231	0.782
	rs2299850	0.063	0.056	0.652	0.068	0.720
	rs9461718	0.144	0.161	0.472	0.157	0.567
	rs2075789	0.081	0.139	**0.002**	0.097	0.485

Abbreviations: MAF, minor allele frequency.

^a^Case 1: idiopathic infertile men with azoospermia or oligozoospermia.

^b^Case 2: idiopathic infertile men with normal sperm count.

Data in bold highlights the statistic significant results.

^c^False Discovery Rate (FDR) corrected *P*-value.

Data in *boldface *represent *P *< 0.05.

Logistic regression analyses showed that in the dominant-effect model, significantly increased risks of azoospermia or oligozoospermia were associated with rs4647269 CT/TT (adjusted OR = 1.63, 95% CI: 1.10 to 2.41), rs1059060 GA/AA (adjusted OR = 1.60, 95% CI: 1.17 to 2.18) and rs2075789GA/AA (adjusted OR = 1.83, 95% CI: 1.32 to 2.55), as compared to wild-type homozygous carriers (Table 3). Meanwhile, a significantly increased risk of male infertility with normal sperm count was associated with the rs1059060 GA/AA genotypes (adjusted OR = 1.83, 95% CI: 1.37 to 2.43), as compared to wild-type homozygotes.

**Table 3:** Genotype frequencies of the four SNPs in MMR genes in patients and controls and their associations with male infertility risk

SNP ID	Genotype	Controls (n = 480)	**Case 1**^**a **^**(n = 524)**		Case 2^b ^(n = 768)	
				
		N (%)	N (%)	**OR (95% CI)**^**c**^	N (%)	**OR (95% CI)**^**c**^
*MLH1*						
rs4647269	CC	431 (90.5)	444 (85.5)	Reference	665 (86.6)	Reference
	CT	45 (9.4)	72 (13.9)	1.56 (1.05 to 2.32)	94 (12.2)	1.34 (0.92 to 1.96)
	TT	0 (0.0)	3 (0.6)	NA	4 (0.5)	NA
	CT/TT	45 (9.4)	75 (14.4)	1.63 (1.10 to 2.41)	98 (12.7)	1.39 (0.93 to 2.01)
*P*_trend_				0.009		0.044
*PMS2*						
rs1059060	GG	393 (82.6)	387 (74.6)	Reference	534 (69.5)	Reference
	GA	79 (16.6)	112 (21.6)	1.43 (1.03 to 1.96)	197 (25.6)	1.82 (1.36 to 2.44)
	AA	4 (0.8)	20 (3.8)	5.03 (1.70 to 14.84)	31 (4.0)	5.65 (1.98 to 16.15)
	GA/AA	83 (17.4)	132 (25.4)	1.60 (1.17 to 2.18)	228 (29.6)	1.83 (1.37 to 2.43)
*P*_trend_				0.0003		< 0.0001
*MSH5*						
rs2075789	GG	401 (85.7)	392 (76.4)	Reference	626 (83.2)	Reference
	GA	58 (12.4)	99 (19.3)	1.73 (1.22 to 2.47)	106 (14.1)	1.16 (0.82 to 1.63)
	AA	9 (1.9)	22 (4.2)	2.48 (1.13 to 5.46)	20 (2.6)	1.41 (0.63 to 3.12)
	GA/AA	67 (14.3)	121 (23.6)	1.83 (1.32 to 2.55)	126 (19.7)	1.19 (0.86 to 1.64)
*P*_trend_				0.0002		0.224

^a^Case 1: idiopathic infertile men with azoospermia or oligozoospermia.

^b^Case 2: idiopathic infertile men with normal sperm count.

^c^Adjustment for age, smoking status and alcohol use.

### Association between MMR polymorphisms and sperm DNA fragmentation

**Figure 1:**
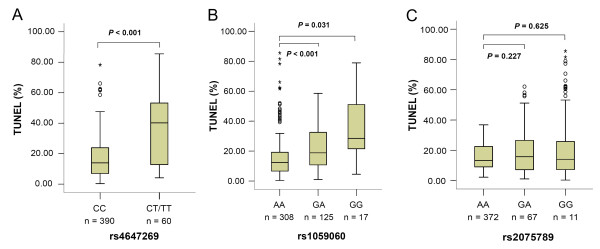
**Box-and-whisker plots of sperm DNA fragmentation for different genotypes**. The boxes represent the 25^th ^and 75^th ^percentiles; whiskers are lines extending from each end of the box covering the extent of the data on 1.5 × inter-quartile range. The median value is denoted as the line that bisects the boxes. Circles and asterisks represent the outlier values. Significant differences were measured by multiple linear regression.

Considering the importance of the MMR pathway in maintenance of DNA integrity, we further evaluated the effects of these three SNPs on sperm DNA fragmentation. In the present study, semen samples were pre-treated with cryopreservation prior to TUNEL analyses. However, it has been demonstrated that the process of cryopreservation can lead to an increase in oxidative stress and percentage DNA fragmentation [27]. To determine whether the results of the TUNEL analyses were profoundly influenced by cryopreservation in our study, 10 semen samples were pre-treated with or without cryopreservation prior to TUNEL analyses. As shown in Additional file 3 (Table S3), modest but significant elevated levels of sperm DNA fragmentation were induced by cryopreservation (*P *= 0.001). However, all the semen samples undergo the same cryopreservation process, thus we believe that the effect of cryopreservation, if any, is unlikely to be substantial. The non-normal distribution of sperm DNA damage levels and sperm concentration were natural log (ln) transformed for further association studies (skewness-kurtosis tests *P *> 0.05). After adjustment for age, smoking, alcohol use and length of abstinence, we found that subjects who carried the rs4647269 CT/TT genotypes displayed markedly higher levels of sperm DNA fragmentation compared with the CC homozygotes (mean ± S.D., 11.82% ± 2.66% vs. 26.58% ± 1.97%; *P *< 0.001) (Figure 1A). Moreover, a gradual increase in sperm DNA fragmentation was found among the three *PMS2 *rs1059060 subgroups (mean ± S.D., 12.30% ± 2.72%, 17.99% ± 2.27%, and 24.78% ± 1.70% for GG, GA and AA, respectively; *P*_trend _< 0.001) (Figure 1B). However, no significant difference was observed for the *MSH5 *rs2075789 (Figure 1C).

### Effects of the PMS2 Ser775Asn polymorphism on MLH1 and PMS2 interaction

**Figure 2:**
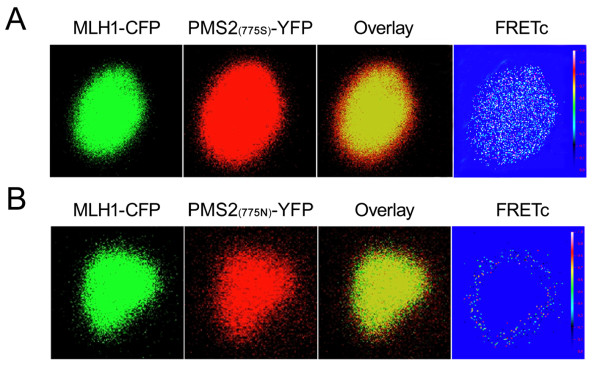
**FRET imaging of MLH1 and PMS2 interaction in live HEK293T cells**. Images of CFP-tagged (green) and YFP-tagged (red) constructs when transiently expressed in HEK293T cells. Co-localization of co-expressed constructs is shown as yellow in overlay images. The pseudocoloured images represent FRET signals corrected for any bleed-through using the micro-FRET method (FRETc). **A**: Co-localization (overlay) and direct interactions (FRETc) between MLH1-CFP + PMS2 (wt)-YFP were detected in the nucleus. **B**: Cells co-expressing MLH1-CFP + PMS2 S775N-YFP showed good co-localization of fluorescent signals but little detectable FRETc signal in the nucleus.

The *PMS2 *Ser775Asn polymorphism (rs1059060) was potentially located within the MLH1-PMS2 interacting domain. Therefore, we examined whether *PMS2 *Ser775Asn polymorphisms influence binding between MLH1 and PMS2. HEK293T cells were transiently co-transfected with plasmids encoding the MLH1 (amino acids 506-756) and wild-type or genetic variants of PMS2 (amino acids 675-850). The schematic diagram of the FRET assay is summarized in Additional file 4 (Figure S1). By confocal fluorescence detection, we found that there was a weak interaction between MLH1 and PMS2-775Asn proteins, for little FRETc was detected in cells co-expressing CFP-MLH1 and YFP-PMS2-775Asn plasmid (Figure 2B). Cells co-expressing CFP-MLH1 + YFP-PMS2-775Ser had a four-fold increase in FRETc values (0.031 ± 0.013, n = 12; 0.008 ± 0.005, n = 12; *P *< 0.001) (Figure 2A). This result suggested that the *PMS2 *Ser775Asn polymorphism could significantly influence the interaction between MLH1 and PMS2.

**Figure 3:**
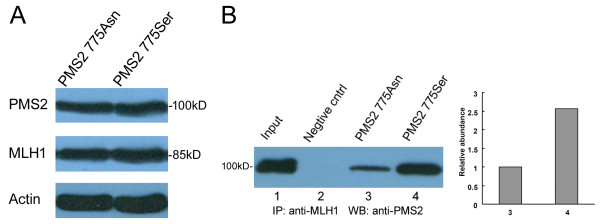
**Interaction studies between hMLH1 and hPMS2 variants**. **A**: Western blot of total protein extracts (50 μg each) from HEK293T cells transfected with pcDNA3.1-MLH1 and either wild-type pcDNA3.1-PMS2 (775Ser) or pcDNA3.1-PMS2 (775Asn) variants. β-actin was used as controls. **B**: The lysates of cells co-expressing the two plasmid were immunoprecipitated with anti-MLH1 N-20 antibody, and then detected with anti-PMS2 (A16-4) antibody. Western blot signals were quantified employing Quantity-One software.

We also used a co-immunoprecipitation assay to detect the effects of *PMS2 *variants on the MLH1 and PMS2 interaction. Full-length MLH1 and PMS2 775Ser or PMS2 775Asn were constructed and co-transfected into HEK293T cells. Western blot analysis of whole cell lysates showed satisfied transfection efficiency (Figure 3A). The co-immunoprecipitated result also suggested that binding between MLH1 and PMS2-775Ser was more robust compared with binding between MLH1 and the PMS2-775Asn variant (Figure 3B, lane 4 vs. 3) which suggested that mutation of PMS2 significantly attenuated the protein-protein interaction of MLH1 and PMS2.

## Discussion

Accumulating evidence demonstrates that MMR plays a critical role in the maintenance of genetic integrity and participates in the meiotic recombination process [14-16]. Although mutations in MMR genes are considered as potential risk factors for various cancers [28,29], only limited data exist on the potential role of polymorphisms in the MMR genes on male infertility. To our knowledge, this study is the first to provide a comprehensive evaluation of the relationship between polymorphisms in MMR and susceptibility to male infertility in a relatively large sample size. On the basis of analysis of 480 controls and 524 infertility patients with azoospermia or oligozoospermia, we observed that one intronic SNP in *MLH1 *(rs4647269) and two non-synonymous SNPs in *PMS2 *(rs1059060, Ser775Asn) and *MSH5 *(rs2075789, Pro29Ser) were associated with increased susceptibility to poor sperm production.

As an important pathway in the DNA damage repair network, MMR also plays a critical role in the maintenance of genetic integrity. Thus, it would be expected that these three significant SNPs that affect sperm DNA integrity could also modify male infertility risk. Based on a case-control study consisting of 480 controls and 768 patients with normal sperm count, we found that *PMS2 *rs1059060 was significantly associated with male infertility with normal sperm count. Further analysis based on 450 infertile men revealed significant associations of *MLH1 *rs4647269 and *PMS2 *rs1059060 with sperm DNA fragmentation. However, we did not detect any association between *MSH5 *Pro29Ser polymorphisms and sperm DNA damage. This result is explained by the fact that MSH5 is a meiosis-specific protein crucial for reciprocal recombination, and it has no apparent mismatch repair activity because it is missing the appropriate amino acid residues [30].

MLH1 and PMS2 form the MutLα heterodimer that leads to the repair of mismatched DNA through activation of exonuclease-mediated degradation of DNA [31]. Guerrette *et al. *localized the MLH1-PMS2 interaction region to amino acids 506-675 of MLH1 and amino acids 675-850 of PMS2 [32]. It is conceivable that the *PMS2 *Ser775Asn polymorphism could directly impact the integrity of the interaction between MLH1 and PMS2. In the present study, we provided evidence, for the first time, that the *PMS2 *Ser775Asn variant attenuates the interaction of MLH1 and PMS2, as illustrated by FRET and co-immunoprecipitation assays.

The *MSH5 *rs2075789 polymorphism in the coding region of the human *MSH5 *gene leads to a proline to serine alteration and is located within the MSH4-MSH5 interacting domain. To address the effect of the Pro29Ser alteration on the interaction between MSH4 and MSH5, a quantitative yeast two-hybrid analysis has been performed [33]. This alteration causes a moderate but significant reduction in the interactions between both proteins, which could affect the formation of the MSH4-MSH5 heterocomplex. These findings strongly support our molecular epidemiological observation that the MSH5 Pro29Ser polymorphism is associated with a significantly increased risk of azoospermia or oligozoospermia. Supporting evidence also comes from association studies by other investigators. In a recent study of a Chinese population with a small sample size, Xu *et al. *observed a 2.89-fold increased risk of azoospermia or oligozoospermia among the *MSH5 *Pro29Ser allele carriers [34]. In addition, a case-control study including 41 women with premature ovarian failure and 39 controls suggested that there is a correlation between the MSH5 Pro29Ser polymorphism and premature ovarian failure in women [35].

Another SNP associated with risk in our study (rs4647269) is intronic. However, SNP rs4647269 tags SNP rs9852810 (r^2 ^= 1, *D*' = 1), which was associated with prostate cancer risk and prostate cancer recurrence [36]. Because both of these two SNPs are located in the intron of the *MLH1 *gene, it is uncertain which one of these two variants causes increases in male infertility risk. To identify additional SNPs that could be associated with male infertility risk that may be in high linkage disequilibrium (LD) with these two sites, we screened all of the common variants (with MAF > 0.05) within an approximately 20 kb-long region surrounding these two sites (approximately 10 kb upstream and approximately 10 kb downstream of these loci) based on the CHB HapMap data resource. We found that rs4647269 is in complete LD with SNP rs1046512, which is located approximately 2.5 kb upstream of start codon of *MLH1*. Therefore, it is highly likely that the rs1046512 SNP near the 5' region of the *MLH1 *gene may be the causal variant.

Another interesting finding was that smoking was associated with increased risk of male infertility. Although the effects of tobacco cigarette smoke on male reproduction are somewhat inconclusive, a number of studies have shown higher incidences of abnormal sperm morphology [37,38] and decreased sperm motility concentration in men who smoke [39,40]. A meta-analysis [41], including 27 studies, indicated that cigarette smoking is associated with a 13% reduction in sperm concentration, a 10% reduction of sperm motility, and a 3% reduction of morphologically normal sperm. Furthermore, fluctuation in reproductive hormone levels have been documented in male smokers [42,43]. However, the mechanism(s) of these changes, if any, remains unclear.

Of note, like all case-control studies, selection bias may exist and might influence interpretation of the results. However, we believe that potential confounding bias might have been minimized by matching the controls to the cases on age and by further adjustment for the confounding factors in statistical analyses. In addition, the fact that genotype frequencies of all SNPs in our controls fit Hardy-Weinberg equilibrium and were similar to those obtained from the HapMap Project further supports the randomness of our control selection. We believe that the selection bias, if any, is unlikely to be substantial.

## Conclusions

The present study extends the previous understanding of the MMR polymorphisms and their effects on the risk of idiopathic azoospermia or oligozoospermia by further evaluating the contribution of these polymorphisms in relation to sperm DNA fragmentation. These novel findings might be helpful in improving the understanding of the role of genetic variation in susceptibility to reduced sperm DNA integrity and in providing diagnostic implications for assisted reproduction success rates. Although, these three SNPs (rs4647269, rs1059060 and rs2075789) associated with risk in our study are significantly higher for some variants in the patient group, the actual rates are quite low and would potentially account for a low percentage of infertility. However, it is important to know that genetic variants associated with common complex diseases like male infertility are only "one piece of the puzzle" making up an individual's overall risk for disease. It is highly likely that the genetic risk for developing male infertility is influenced by the additive effects of many different genetic variants and other risk factors. So, further research is required to define their interactions with other susceptibility alleles and environmental factors can lead to a substantial increase in male infertility risk, especially when exposed to certain dietary and lifestyle habits.

## Abbreviations

MMR: mismatch repair; SNP: single-nucleotide polymorphism; TUNEL: Tdt-mediated dUTP nick end labelling; FDR: False Discovery Rate; FRET: fluorescence resonance energy transfer

## Competing interests

The authors declare that they have no competing interests.

## Authors' contributions

GXJ conceived and designed the experiments, performed the experiments, analyzed the data and drafted the manuscript. YZ contributed to the experimental design and data analysis. CH contributed to the sample preparation, genotyping and drafted the manuscript. YL contributed to the FRET and Co-IP assays and drafted the manuscript. XRW, AHG and YZ conceived and designed the experiments, and revised the manuscript. All authors read and approved the final manuscript.

## Pre-publication history

The pre-publication history for this paper can be accessed here:

http://www.biomedcentral.com/1741-7015/10/49/prepub

## Supplementary Material

Additional file 1 **Additional file 1**. Primer Sequences for SNPstream Genotyping and TaqMan analysis. Click here for file

Additional file 2 **Additional file 2**. Information on genotyped tSNPs of the MMR genes evaluated in this study. Click here for file

Additional file 3 **Additional file 3**. Effect of cryopreservation on sperm DNA fragmentation. Click here for file

Additional file 4 **Additional file 4 (Figure S)**. Schematic diagram of the recombinant plasmids containing the potential MLH1-PMS2 interaction domain in the fluorescence resonance energy transfer (FRET) assay. Click here for file
