# Supplementary material for: Common variants in mismatch repair genes associated with increased risk of sperm DNA damage and male infertility
Source: BMC Med. 2012 May 17;10:49. doi: 10.1186/1741-7015-10-49 (PMC3378460; doi:10.1186/1741-7015-10-49)
Supplement: Additional file 1 — Primer Sequences for SNPstream Genotyping and TaqMan analysis. [file 1741-7015-10-49-S1.DOC]

Supplementary Table 1. Primer Sequences for SNPstream Genotyping and TaqMan analysis

| SNP ID |  | Amplification PCR primer (5'-3') |  | PCR product (bp) |  | Extension Primer (5'-3') |
| --- | --- | --- | --- | --- | --- | --- |
| rs1799977 (A>G) |  | F: TGATGTTAGGACACTACCCAATG  R: AAGATTTTTTTATATAGGTTATCGACATACC |  | 102 |  | GGCTATGATTCGCAATGCTTCTCAACCGTGGACAATATTCGCTCC |
| rs4647269 (C>T) |  | F: AAAAAAATAAGACTAAAATCAGGGG  R: ATTGATAAAGTCACAACCACTTTTC |  | 171 |  | AGGGTCTCTACGCTGACGATCCTGTGTTGCTGTAGACAAGGATAC |
| rs3815383 (C>T) |  | F: TTCTGTAAATGCACAAAATAAGATAATG  R: ACCCAGCAAAGGTATTTAAAATTT |  | 157 |  | GTGATTCTGTACGTGTCGCCTGTTTCTCAAAGTCCCGAGCTCCAC |
| rs2286680 (G>A) |  | F: TGAGGATTTATCAGGAAGGGTA  R: ATGMGTCATCAAAGAACCAAAC |  | 131 |  | GGCTATGATTCGCAATGCTTGGAGAGCTGCAACACATCAGTTTTG |
| rs11769380 (C>T) |  | F: AATTTGTTCGAAGAGTAATAACAAAAC  R: TAAAAGACAGTGAGACAGAGAGCA |  | 153 |  | CGTGCCGCTCGTGATAGAATAAATCTTAAGATCATTTATGGTTTT |
| rs1059060 (G>A) |  | F: TTTTAGCTCCAGTCACTGAAAG  R: CATGAAGATCAGTTCATCGAC |  | 98 |  | GGATGGCGTTCCGTCCTATTCTGATTTCCTTGCCAACTAGTAAAA |
| rs2228006 (G>A) |  | F: TGAGGATTTATCAGGAAGGGTA  R: ATGMGTCATCAAAGAACCAAAC |  | 131 |  | GGCTATGATTCGCAATGCTTGGAGAGCTGCAACACATCAGTTTTG |
| rs175049 (C>T) |  | F: TAAAACTTGAGAGATGAGATGTAAAATG  R: CCATAGGAACCTTCACTTCTCA |  | 99 |  | AGAGCGAGTGACGCATACTAGCCATTCTTGAGAGTTCCTTTTTCT |
| rs1021462 (C>T) |  | 5’-ATTATTTCTTCTGTCCCTTCTTCAC-3’ (sense)  5’-ATGCTTGAGCCCAGGAAT-3’ (antisense) |  | 120 |  | 5’-AGATAGAGTCGATGCCAGCTCTTTTTTTTGAAACAAGGTCTCACT-3’ |
| rs3749953 (A>G) |  | 5’-CTCAGTGTACAAAGTGGCCAG-3’ (sense)  5’-ACACATCTTTGGAGCCAATT-3’ (antisense) |  | 121 |  | 5’-GACCTGGGTGTCGATACCTAAGGTGTGCCCCATCCCTCATCTCAC-3’ |
| rs1150793 (T>C) |  | 5’-TGACATCATCTCTAAACAGAGTGG-3’ (sense)  5’-ATGCTGATTACCTGGAGATGAC-3’ (antisense) |  | 141 |  | 5’-GTGATTCTGTACGTGTCGCCTACCTCACCTCCCGCTGCAATGTGT-3’ |
| rs707938 (T>C) |  | 5’-ATGCCCCCACATCTTTGT-3’ (sense)  5’-TAGATTGGTCTCCTCACCAAATA-3’ (antisense) |  | 102 |  | 5’-AGATAGAGTCGATGCCAGCTACTTTCTGAGCCTTGTTCAGCTACA-3’ |
| rs3115672 (G>A) |  | 5’-TTTGACCCTCCCTTTGTCC-3’ (sense)  5’-TCCGCCCACTGCAGACAT-3’ (antisense) |  | 93 |  | 5’-AGAGCGAGTGACGCATACTACACATTCTGTGGAGTTGGGCACAAA-3’ |
| rs3117572 (G>A) |  | 5’-AAATGTAAACGCACTGGTGAC-3’ (sense)  5’-ATTACCTGGAGATGACCTCAGA-3’ (antisense) |  | 152 |  | 5’-CGACTGTAGGTGCGTAACTCCCCCTACCTCACCTCCCGCTGCAAT-3’ |
| rs2075789 (G>A) |  | 5’-ATTCAAGTTTCTACTCCCCTCAG-3’ (sense)  5’-ACACCGCAGGGACCGAGA-3’ (antisense) |  | 144 |  | 5’-ACGCACGTCCACGGTGATTTTCGGCCTCCCTGGGGCCCGGCACTG-3’ |
| Taqman assay | | | | | | |
| rs13712 (A>C) |  | F: AACCCAACCTCACTAAACT  R: ACATCCCTTTGTTCCTTT |  | 153 |  | FAM-CTGCAGCAATCCATGCCT-MGB  HEX-CTGCAGCACTCCATGCCT-MGB |
| rs1540354 (T>A) |  | F: CAAAGTGCTGAGAACCACGGC  R: CCTGGAGTCTTCGCCTAGCT |  | 113 |  | FAM-CAATAAATCTTCCTGTATAA-MGB  HEX-CAATAAATCTACCTGTATAA-MGB |
| rs7156586 (T>A) |  | F: TTCTCAATATGGGCTTTC  R: CCTTTAGTGACCTTGTCC |  | 99 |  | FAM-CTAAAAGGGCTCTGTTGCAC-MGB  HEX-CTAAAAGGGCACTGTTGCAC-MGB |
| rs707939 (G>T) |  | 5’-CCCACCCTAGAGGGAGCTTA-3’ (sense)  5’-TGTCATCTACATCCCTCTGGTG-3’ (antisense) |  | 181 |  | 5’-FAM-CCCAAAAGACATCTG-MGB-3’  5’-HEX-CCCAAAATACATCTGA-MGB-3’ |
| rs9461718 (A>C) |  | 5’- GGCACCTCGCATGGCTT-3’ (sense)  5’-GGCAGGTAAGAATAGAGGCGG-3’ (antisense) |  | 103 |  | 5’-FAM-ATCAAGATAGATGAACCC-MGB-3’  5’-HEX-ATCAAGAGAGATGAACC-MGB-3’ |
| rs2299850 (G>A) |  | 5’- AATGGAATCAGACAGGGCAT’ (sense)  5’-GCTGGGATTATAGGCGTGAG-3’ (antisense) |  | 198 |  | 5’-FAM-ACCTCTAACGTACAAAA-MGB-3’  5’-HEX-AATACCTCTAATGTACAAAA-MGB-3’ |

F: forward primer; R: reverse prime
