# Supplementary material for: Common variants in mismatch repair genes associated with increased risk of sperm DNA damage and male infertility
Source: BMC Med. 2012 May 17;10:49. doi: 10.1186/1741-7015-10-49 (PMC3378460; doi:10.1186/1741-7015-10-49)
Supplement: Additional file 2 — Information on genotyped tSNPs of the MMR genes evaluated in this study. [file 1741-7015-10-49-S2.DOC]

Supplementary Table 2. Information on genotyped tSNPs of the MMR genes evaluated in this study

| Gene | SNP ID | Nucleotide  change | Function | MAF* | *P* value for  HWE test | % Genotyped  rate |
| --- | --- | --- | --- | --- | --- | --- |
| *MLH1* |  |  |  |  |  |  |
|  | rs1799977 | A > G | Exon | 0.044 | 0.781 | 99.7 |
|  | rs4647269 | C > T | Intron | 0.056 | 0.493 | 99.3 |
|  | rs1540354 | T > A | Intron | 0.344 | 0.492 | 98.5 |
| *PMS2* |  |  |  |  |  |  |
|  | rs3815383 | C > T | Intron | 0.310 | 0.581 | 99.1 |
|  | rs2286680 | G > A | Intron | 0.089 | 0.326 | 99.4 |
|  | rs11769380 | C > T | Intron | 0.414 | 0.603 | 96.9 |
|  | rs1059060 | G > A | Exon | 0.298 | 0.989 | 99.1 |
|  | rs2228006 | G > A | Exon | 0.078 | 0.142 | 99.5 |
| *MLH3* |  |  |  |  |  |  |
|  | rs13712 | A > C | Exon | 0.122 | 0.558 | 98.7 |
|  | rs7156586 | G > A | Intron | 0.250 | 0.054 | 98.6 |
|  | rs175049 | C > T | 3' UTR | 0.073 | 0.624 | 99.3 |
| *MSH4* |  |  |  |  |  |  |
|  | rs1021462 | C > T | Intron 19 | 0.433 | 0.558 | 98.5 |
| *MSH5* |  |  |  |  |  |  |
|  | rs3749953 | A > G | Intron 9 | 0.200 | 0.656 | 99.0 |
|  | rs1150793 | T > C | Intron 10 | 0.089 | 0.853 | 96.1 |
|  | rs707939 | G > T | Intron 15 | 0.311 | 0.427 | 99.9 |
|  | rs707938 | T > C | Exon 22 | 0.344 | 0.492 | 98.5 |
|  | rs3115672 | G > A | Exon 19 | 0.378 | 0.452 | 98.2 |
|  | rs3117572 | G > A | Intron 10 | 0.239 | 0.024 | 98.6 |
|  | rs2299850 | C > T | Intron 10 | 0.071 | 0.142 | 99.5 |
|  | rs9461718 | A > C | Intron 18 | 0.122 | 0.611 | 100 |
|  | rs2075789 | G > A | Exon 2 | No report | 0.388 | 97.8 |

Abbreviations: MAF, minor allele frequency; HWE, Hardy-Weinberg equilibrium.

*Minimum allele frequency in the general Han Chinese, as reported in dbSNP database.
