# Supplementary material for: Common variants in mismatch repair genes associated with increased risk of sperm DNA damage and male infertility
Source: BMC Med. 2012 May 17;10:49. doi: 10.1186/1741-7015-10-49 (PMC3378460; doi:10.1186/1741-7015-10-49)
Supplement: Additional file 3 — Effect of cryopreservation on sperm DNA fragmentation. [file 1741-7015-10-49-S3.DOC]

Supplementary Table 3. Effect of cryopreservation on sperm DNA fragmentation.

|  | Sperm DNA fragmentation (%) | | | | | | | | | | |  |
| --- | --- | --- | --- | --- | --- | --- | --- | --- | --- | --- | --- | --- |
| No. | 1 | 2 | 3 | 4 | 5 | 6 | 7 | 8 | 9 | 10 | mean±SE | *P*-value^a^ |
| cryopreservation | 35.8 | 24.8 | 15.2 | 26.7 | 35.8 | 43.7 | 32.5 | 19.2 | 27.7 | 17.8 | 27.9±2.9 | 0.001 |
| Fresh sample | 32.5 | 23.9 | 15.1 | 23.3 | 31.7 | 38.3 | 31.5 | 17.1 | 25.5 | 16.0 | 25.5±2.5 |  |

^a^The t-test (two-tailed) testing the difference between means of sperm DNA fragmentation for two groups.
