# Supplementary figures and images for: Common variants in mismatch repair genes associated with increased risk of sperm DNA damage and male infertility
Source: BMC Med. 2012 May 17;10:49. doi: 10.1186/1741-7015-10-49 (PMC3378460; doi:10.1186/1741-7015-10-49)

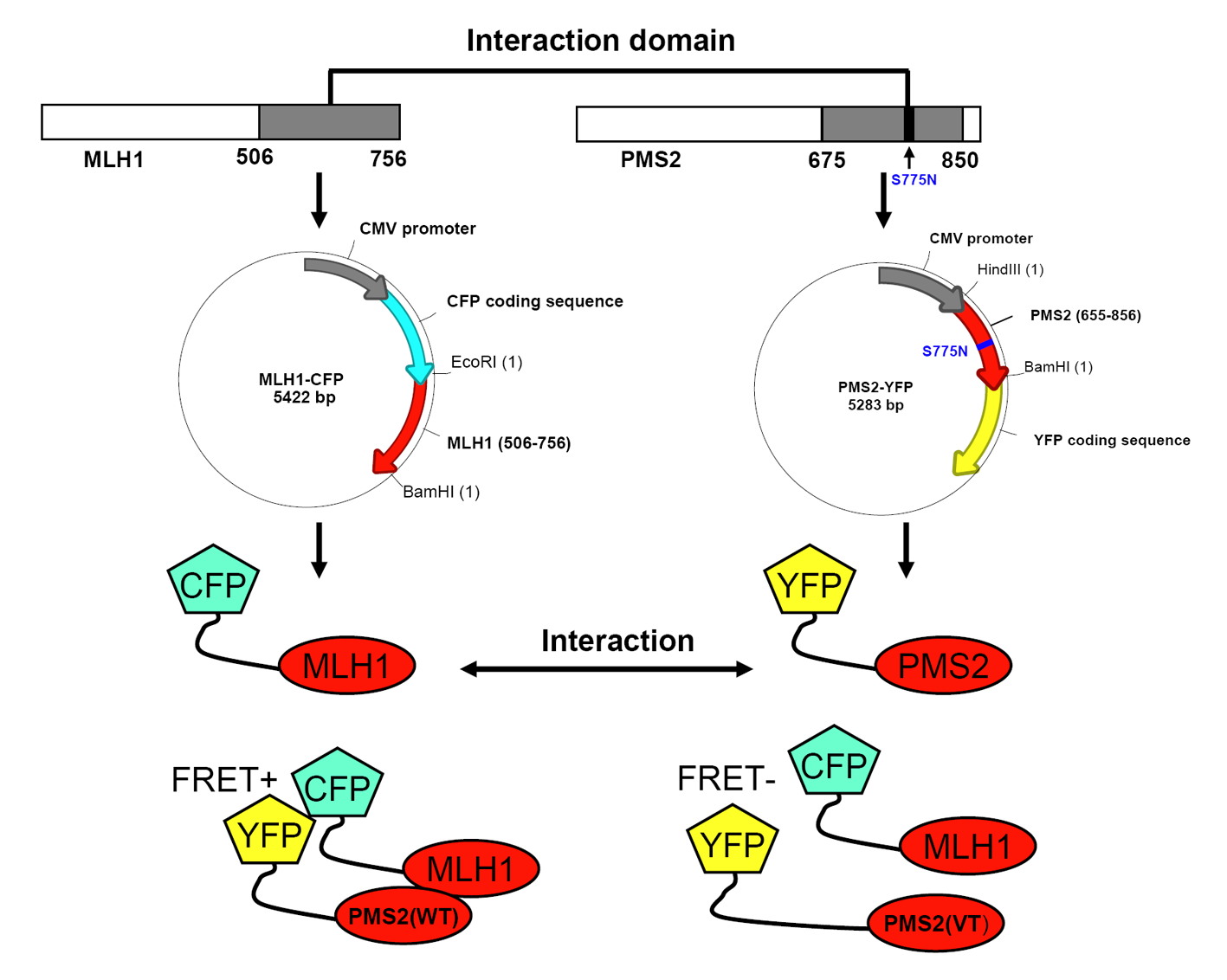

Supplement: Additional file 4 — (Figure S). Schematic diagram of the recombinant plasmids containing the potential MLH1-PMS2 interaction domain in the fluorescence resonance energy transfer (FRET) assay. [file 1741-7015-10-49-S4.TIFF]
